# Electric Field-Controlled Crystallizing CaCO_3_ Nanostructures from Solution

**DOI:** 10.1186/s11671-016-1338-4

**Published:** 2016-03-01

**Authors:** Jian Quan Qi, Rui Guo, Yu Wang, Xuan Wen Liu, Helen Lai Wah Chan

**Affiliations:** School of Materials Science and Natural resources, Northeastern University at Qinhuangdao, Qinhuangdao, 066004 Hebei Province People’s Republic of China; Department of Applied Physics and Materials Research Center, The Hong Kong Polytechnic University, Hung Hom, Hong Kong

**Keywords:** CaCO_3_, Nanoflower, Electric field

## Abstract

The role of electric field is investigated in determining the structure, morphology, and crystallographic characteristics of CaCO_3_ nanostructures crystallized from solution. It is found that the lattice structure and crystalline morphology of CaCO_3_ can be tailed by the electric field applied to the solution during its crystallization. The calcite structure with cubic-like morphology can be obtained generally without electric field, and the vaterite structure with the morphology of nanorod is formed under the high electric field. The vaterite nanorods can be piled up to the petaliform layers. Both the nanorod and the petaliform layer can have mesocrystal structures which are piled up by much fine units of the rods with the size of several nanometers. Beautiful rose-like nanoflowers can be self-arranged by the petaliform layers. These structures can have potential application as carrier for medicine to involve into metabolism of living cell.

## Background

Electric field is ubiquitous in nature. It causes the phenomena as great as thunder and lightning or even as fine as the electrostatic interaction among atoms or molecules. It was proposed that the earliest organic compound to biomolecule was produced in the ancient sea on the earth under high electric field, i.e., thunder and lightning [1]. The carbon nanotube was also firstly synthesized by point discharge between two graphite electrodes under high electric field [2]. Now, electric field offers a facility for various controlling technically. Electrospinning is one of the electric field controlled techniques that has gained tremendous attention in the last decade [3, 4] as polymer-processing method for polymer nanofibers [5] and tissue engineering scaffold [6], even as well as to metals and ceramics [7]. Electric field-controlled sintering is also an important method for the fabrication of a textured ceramics. However, the chemical reaction and the crystallization of nanostructures from solution under the electric field are not focused enough. Both of them are associated with the electrostatic force, and thus, the investigation to the chemical reaction or crystallization in solutions under a certain electric field will be amazing.

CaCO_3_ is one of the standard model systems because of its abundance in nature, participation in the formation of a certain geologic structure as Karst cave, association with biological calcium metabolism, its important industrial applications. Crystallization and morphological control of CaCO_3_ have attracted extensive attention for decades [8–23]. Much of them focus their attention on surfactant controls. Our study is inspired by the formation of the earliest biomolecule under thunder and lightning, also electrospinning in technology. The polymorphy and morphology of CaCO_3_ nanostructures synthesized under the high electric field from solution is investigated and found that they are much different from those without electric field.

## Methods

**Fig. 1:**
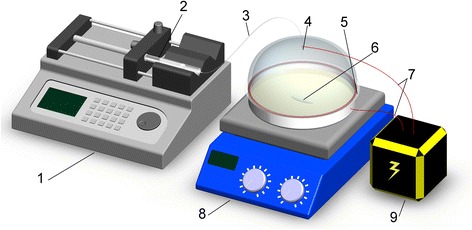
The illustration of the system for synthesis of nanostructures under electric field: *1* ejector jet pump, *2* syringe, *3* perfusion tube, *4* entry needle, *5* stainless steel vessel, *6* magnetic stirrer, *7* electric wire, *8* magnetic stirring apparatus, and *9* high voltage power

The synthesis system is illustrated in Fig. 1 as our patent described [24]. In a typical synthetic process, 3.52 g Ca(Ac)_2_ (BDH Chem. Ltd., UK) was dissolved in 10 ml absolute ethanol (International Laboratory, USA) and then transferred into a syringe as injecta. 2.76 gK_2_CO_3_ (BDH Chem. Ltd., UK) was dissolved in 200-ml deionized water in a stainless steel vessel as a base solution. The vessel was linked to the ground of high voltage power serving as a counter electrode. The syringe was fixed on the ejector jet pump. The syringe nozzle was linked to a perfusion tube, which the other end was fixed on an entry needle. The entry needle simultaneously served as an electrode, to which a high electric field of 0–1000 kV/m can be applied, and the distance to the counter electrode is 10–25 cm in our laboratory systems. The injecta solution was injected into the base solution which was stirred quickly by magnetic stirrer. In our experiments, 0 and 8 kV were employed and perfusion flow was fixed at 80 μl/min. After the completion of the perfusion, obtained white slurry was filtered and washed several times by deionized water. Through the vacuum drying, the white as-prepared powders were checked by the X-ray diffraction (XRD, a Philips Diffractometer, X’Pert-Pro MPD), field emission scanning electron microscopy (FE-SEM, JSM6335F NT), and transmission electron microscopy (TEM, a JEOL TEM, JSM2010).

## Results and Discussion

CaCO_3_ has three anhydrous crystallographic polymorphs: vaterite (the least stable), aragonite, and calcite (the most stable). Vaterite particles do not show well-defined morphologies and usually aggregate into spherical particles. The vaterite form can crystallize in either an orthorhombic or a hexagonal structure. With the aids of surfactants and/or templates, hollow vaterite spheres, micro floret, hexagonal prism vaterite crystalline powders, etc. could be synthesized. There is seldom aragonite in nature. It may be associated with the activity of the creatures on the earth. Among the three polymorphs of CaCO_3_, calcite would be crystallized generally from the solution without outside disturbance because it is the most thermodynamic stable phase.

**Fig. 2:**
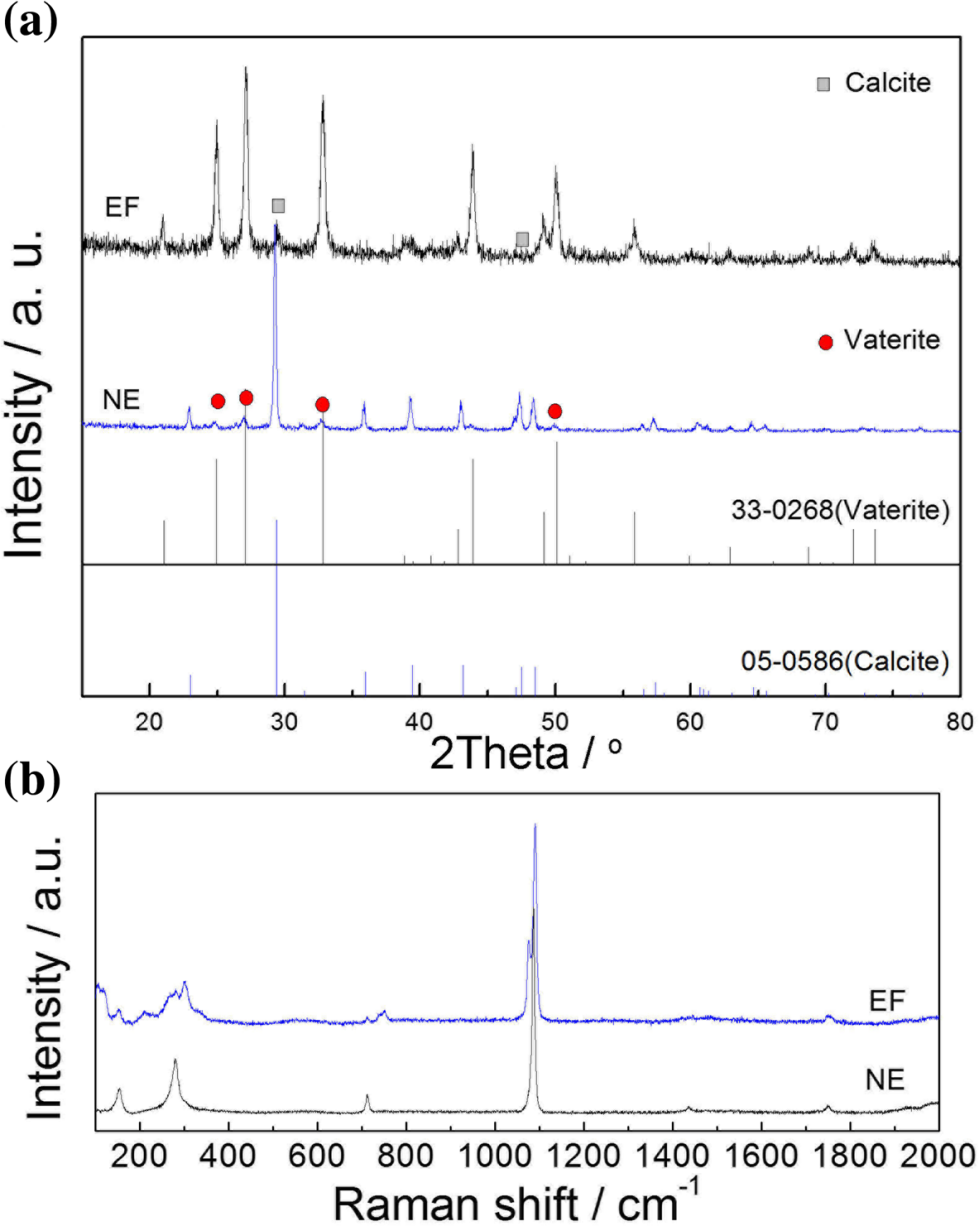
The XRD pattern and Raman spectra of CaCO_3_ synthesized under electric field (EF) and electric free (NE). **a** XRD pattern. **b** Raman spectra

To clear investigate the effect of electric field on the crystallization of CaCO_3_, two comparable experiments have been done. In our study, both the precipitation of CaCO_3_ chemical reactions under 8 kV (~800 kV/mm) electrical field and that under electrical field free (0 kV/mm) were completed in our instrument as described above and named as electric field (EF) sample and electric free (NE) sample, respectively. The XRD patterns are shown in Fig. 2a. It is obviously shown that the main crystal phase of the sample under electric field free is calcite (#pdf:05-0586) as expected. Comparably, the crystal phase of the sample which was fabricated under 8 kV electric field is mainly vaterite (#pdf:33-0268). It is confirmed that vaterite can be synthesized under high electric field, and calcite can be obtained under the condition of electric field free preferentially. In the experiments, there is a little calcite in EF sample except for vaterite as the main phase because applied electric field is not high enough. In comparison with the sample of NE, a little of vaterite are produced due to electrostatic force of the surface tension of the solution which solved acetic salt as surfactant.

The vibrational spectroscopy as Raman spectroscopy is sensitive to the structural transformation; the distortions and defects of lattice cell at the molecular level can be detected [25]. It is a paradigm that the tetragonality of BaTiO_3_ can be identified by the Raman spectrum, and the fine distinction on phase structures can be distinguished [26]. It is also a powerful tool to determine the polymorphs of CaCO_3_ crystals [27–29]. Figure 2b shows the Raman spectrums of sample EF and NE which representatively identified as vaterite and calcite as the literatures documented [27, 29]. The strongest bands of calcium carbonate polymorphs at around 1100 cm^−1^ overlap, but vaterite has double peaks whereas calcite has mono one. The most intense Raman bands are observed in the low frequency region (50–400 cm^−1^), which corresponds to the lattice mode vibrations. In these bands, the characteristic peaks of calcite are strong and narrow, whereas the vaterite bands are large and overlap at 150 and 281 cm^−1^ with calcite. The modes of wide band around 110 and 300 cm^−1^ are the characteristic vibration of vaterite lattice as shown as Fig. 2b.

**Fig. 3:**
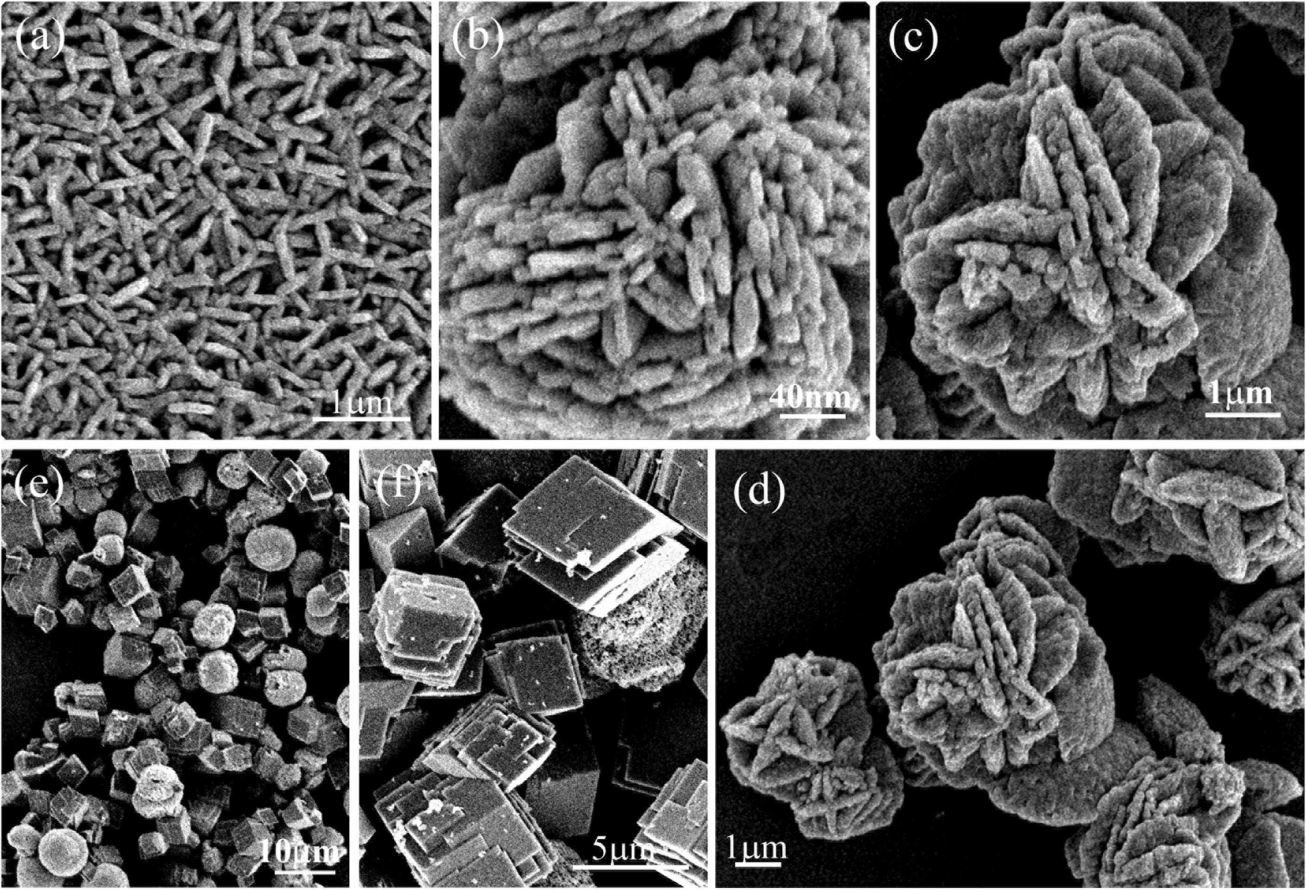
SEM of the samples, EF (**a**–**d**) and NE (**e**, **f**)

The microstructures of EF sample by SEM are shown in Fig. 3a–d in series. The nanorods with the size of 500 nm in length and 50 nm in diameter are apparently shown in Fig. 3a, which synthesized under the electric field. The nanorods can assemble to petaliform layers as some as shown in Fig. 3b spontaneously. Figure 3b clearly shows the media products of the evolution of nanoflowers. It is amazing that these petaliform layers can assemble further to beautiful rose-like nanoflowers as shown in Fig. 3c, d. The petals of the rose are clearly composed by nanorods in these pictures. All of these reveal the beauty of the crystallography.

The microstructures of NE sample are shown in Fig. 3e, f. There are two morphologies in Fig. 3e, cube and sphere inspect of two phases indexed from XRD pattern, calcite and vaterite. From the point of view of crystallization characteristics of the polymorph, the cube is assigned as calcite and sphere as vaterite. Here, the sphere-like morphology of vaterite is much different from that as flower-like in EF sample, although they both have the same phase as vaterite. The calcite morphology is clearly shown in Fig. 3f also. The cubes are clearly grown from square layer by layer. It is interesting that the polymorphs can be obtained from the different conditions of the crystallization.

**Fig. 4:**
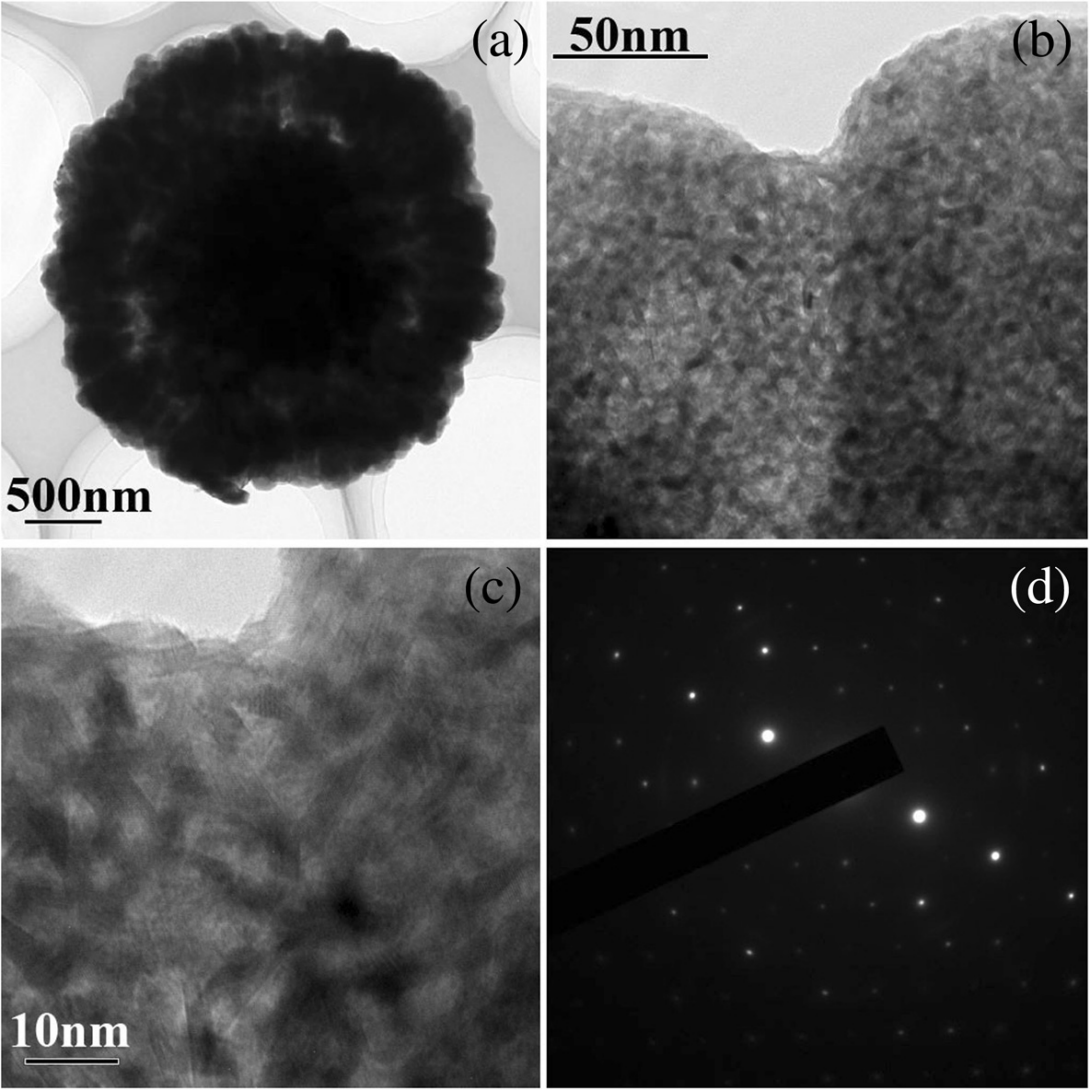
TEM and SEAD pattern of sample EF, (**a**) a petaliform layer, (**b**) a conjunction of two nanorods, (**c**) detail structure of the conjunction, (**d**) SEAD pattern of the conjunction

In order to study the characteristics of the crystallization under the electric field, more details of the sample EF was observed by TEM. Figure 4 has shown a much fine structure of a petaliform layer nanostructure of vaterite. Figure 4a shows a petaliform layer uppiled by a series of nanorods. Figure 4b shows a conjunction of two nanorods. More detail structure of it is shown in Fig. 4c. It is clearly shown that the nanorods and even the conjunction of them are piled up by much fine units of the rods with the size of 10 nm in length and 3 nm in diameter. Much fine pores with the size of about 5 nm among the units are clearly observed. These structures can have some unique characteristics for absorption or/and filter of special molecules with large specific area. Therefore, our samples have the potential application as a carrier for medicine to get involved in metabolism of living cell.

It is amazing when we check the SEAD of the sample. A series of perfect hexagonal single crystal diffraction pattern is revealed in Fig. 4d. Obviously, the petal of rose-like nanostructure is a single crystal from this point of view and is called as mesocrystals. As documented previously, besides conventional crystallization, there are also nonclassical pathways of crystallization via colloidal intermediates and mesoscale transformation [8–11], in which crystalline structures are constructed and/or transformed from larger units instead of single ions. In addition, crystals can be assembled from nanoscopic building units in an almost perfect three-dimensional orientation to form so-called mesocrystals, exhibiting well-faceted outer surfaces. In the case of inorganic minerals [9, 11, 30], these typically fuse to single crystals, likely as a result of high lattice energy, whereas in organic crystals [31], they can be isolated.

**Fig. 5:**
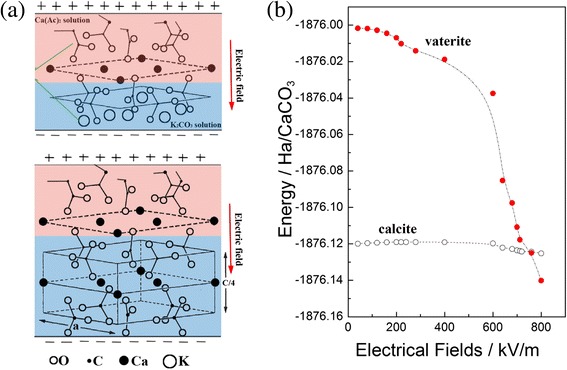
The crystallinegraphic characteristics of CaCO_3_ under the electric field. **a** Illustration of crystallization of CaCO_3_ from solution under electric field [8]. **b** The dependence of energy of calcite and vaterite on electric field strength

Polarized hydroxyapatite ceramics was used to study the effect of surface electric field on crystallization of calcite. It was found the oriented growth of calcite crystals with the rhombohedra {10.4} plane parallel to the polarized hydroxyapatite substrates on both negatively and positively charged surfaces due to the electrostatic force applied to the precipitated ions which accumulated on the surface of the substrates [32]. The electric field plays several roles during crystallization of vaterite nanostructures from the solutions in our study. At first, the high electric field makes the titrant carrying static charge and then splits the titrant to nano-beams by electrostatic forces due to the same charges it taken. Secondarily, it provides an electrical field to make ion orientation in the solution and thus changes the crystallization characteristics of the precipitations, such as orientating depositions or changing morphologies and phases. The crystalline graphic characteristics of CaCO_3_ under the electric field are described in Fig. 5. Crystallization of CaCO_3_ from solution under electric field is illustrated in Fig. 5a. The characteristics of the crystallization of both vaterite and calcite are studied and documented [8]. The formation of a stern layer of Ca counterions favors the oriented nucleation of crystal faces consisting of only Ca atoms and the (0001) face of vaterite fits this criterion. The trigonal planar carbonate anions are oriented parallel to the (0001) face of calcite, whereas in vaterite, they are aligned perpendicular, and this later arrangement is equivalent to the orientation of the carboxylates in conjunction with Ca binding generates a two-layer subunit cell motif of the vaterite structure rather than calcite. In our EF sample, the trigonal plannar carbonate anions are aligned perpendicularly preferentially to a stern layer of Ca counter ions under the electric field, and vaterite crystal can be formed naturally.

To understand the transition between different polymorphs further, density functional theory (DFT) methods can enable itself as an extremely powerful tool for elucidating the energy changing of different polymorphs under electric field [33, 34]. The energy calculations are based on the Kohn-Sham equations which were derived from the Schrödinger equation. All calculations were performed with DMol3 package of Materials Studio. In the first step, the calculation models according to the crystallographic information file of calcite and vaterite were built, respectively. In the next, the energy of them under variable electric field within the density functional theory was calculated, where the exchange-correlation function has been parameterized according to the generalized gradient approximation in the Becke and Lee-yang-Parr function. The basis set “DND” was chosen and DFT Semi-core Pseudo pot was adopted for the core treatment of all atoms in the calculations. The tolerance of SCF is <1.0–6 eV. Satisfactory results were obtained and are shown in Fig. 5b. It can be see that the energy of calcite (−1876.119947 Ha, 1 Ha = 27.2 ev) is lower than that of vaterite (−1876.001730 Ha), whereas the electric field is free, so that calcite is more stable and deposited from solution as the main crystal phase. The energy of both calcite and vaterite decreases with the increase of electric field strength, but the decrease rates of them are much different. The energy of calcite decreases with the electric field strength slowly; on the contrary, that of vaterite decreases speedily and drops down steeply at the electric field strength of about 500 kV/m, till the conversion appears at about 730 kV/m due to the energy of vaterite decreasing more quickly. All of these calculations are well consistent with our experimental results.

## Conclusions

The electric field can be one of the most important facts to determine the polymorphs and morphology of the materials during their synthesis and their crystallization. It is found that the lattice structure and crystalline morphology can be tailed by the electric field which applied to the reaction system during deposition of CaCO_3_ from solution. The calcite structure with cubic-like morphology can be obtained usually during deposition from solution. On the other hand, the vaterite structure with morphology of nanorod is formed under the high electric field. During crystallization of vaterites nanostructures from solutions, the electric field splits the titrant to nano-beams and provides an electrical field to make ions orientation in solution and thus changes the crystallization characteristics of the precipitations. Both calculation and experiments confirmed that the electric field is propitious to the crystallization of vaterite during deposition from solution. The vaterite nanorods we synthesized under high electric field can be self-arranged into beautiful rose-like nanoflowers. The petal of rose-like nanoflowers is detected as mesocrystal, in which crystalline structures are constructed and/or transformed from larger units instead of single ions. Therefore, crystals can be assembled from nanoscopic building units in an almost perfect three-dimensional orientation to form so-called mesocrystals, exhibiting well-faceted outer surfaces.

## Abbreviations

DFT: density functional theory
TEM: transmission electron microscopy
XRD: X-ray diffraction
